# Acute Seizures Occurring in Association With SARS-CoV-2

**DOI:** 10.3389/fneur.2020.576329

**Published:** 2020-11-05

**Authors:** Sean T. Hwang, Ahmad A. Ballout, Usman Mirza, Anup N. Sonti, Arif Husain, Claudia Kirsch, Ruben Kuzniecky, Souhel Najjar

**Affiliations:** Donald and Barbara Zucker School of Medicine at Hofstra/Northwell, North Shore University Hospital, Manhasset, NY, United States

**Keywords:** seizures, SARS-CoV-2, COVID-19, status epilepticus, neurological complication

## Abstract

Seizures are an infrequent and serious neurological complication of SARS-CoV-2 infection, with limited data describing the etiology and the clinical context in which these occur or the associated electrographic and imaging findings. This series details four cases of seizures occurring in patients with COVID-19 with distinct time points, underlying pathology, and proposed physiological mechanisms. An enhanced understanding of seizure manifestations in COVID-19 and their clinical course may allow for earlier detection and improved patient management.

## Introduction

Coronavirus disease 2019 (COVID-19) associated with severe respiratory syndrome coronavirus 2 (SARS-CoV-2) presents with cough, fever, fatigue, and gastrointestinal symptoms. However, neurologic manifestations are reported in a third of patients and include dizziness, headaches, impaired consciousness, neuromuscular injury, changes in taste or smell, stroke, ataxia, or seizures (1).

Two early multicenter studies from China reported seizures in 0.47–0.66% of patients with COVID-19 (1, 2). Subsequent individual cases of seizures associated with COVID-19 were described, both as a presenting feature of COVID-19 and as a complication emerging later in the course of critically ill patients (3–12). Although seizures are uncommon, their occurrence is not unexpected given their association with other human coronavirus infections such as SARS-CoV and MERS reported in the literature (13, 14). There is increasing appreciation of the SARS-CoV-2 pathogenic mechanisms causing neurological injury and seizures.

This paper presents the clinical course and electroencephalographic (EEG) and neuroradiographic features of four cases of acute symptomatic seizure in the setting of SARS-COV-2 infection, highlighting distinct pathological mechanisms by which seizures may occur in the context of the unique and formidable illness of COVID-19.

## Case Presentations

### Case 1

A 48-year-old woman presented to urgent care complaining of fever, cough, vomiting, and malaise. Pneumonia was confirmed on chest X-ray, and she was prescribed with doxycycline and azithromycin. Three days later, she was brought in emergently for progressive confusion. She was febrile and tachypneic, with a blood pressure of 179/87 mmHg. She was alert, though aphasic, with impaired verbal output, naming, comprehension, and repetition. Babinski reflex was positive bilaterally.

The patient then experienced three consecutive generalized tonic–clonic seizures (GTCS) associated with full body stiffening, dilated pupils, foaming at the mouth, and tongue bite up to 6 min apart, concerning for status epilepticus for which she was administered intravenous (IV) lorazepam and levetiracetam with resolution.

Initial laboratories were notable for mild transaminitis (AST 34 μ/L, ALT 53 μ/L) and elevated inflammatory markers (D-dimer 279 ng/ml, ferritin 357 ng/ml, and C-reactive protein 32 mg/ml). The toxicology result was negative. The chest X-ray result revealed bilateral interstitial infiltrates, and non-contrast computed tomography (CT) of the head was unrevealing. The patient was empirically treated for encephalitis with acyclovir and ceftriaxone. Lumbar puncture was traumatic, with the white blood cell count corrected to five per cubic millimeter and elevated protein of 125 mg/dl. The results of cerebrospinal fluid (CSF) gram stain, culture, and polymerase chain reaction (PCR) for herpes were subsequently negative. The nasopharyngeal COVID-19 PCR test result was positive. CSF COVID-19 PCR was unavailable. Continuous EEG was performed postictally, showing severe generalized slowing but no epileptiform activity.

**Figure 1:**
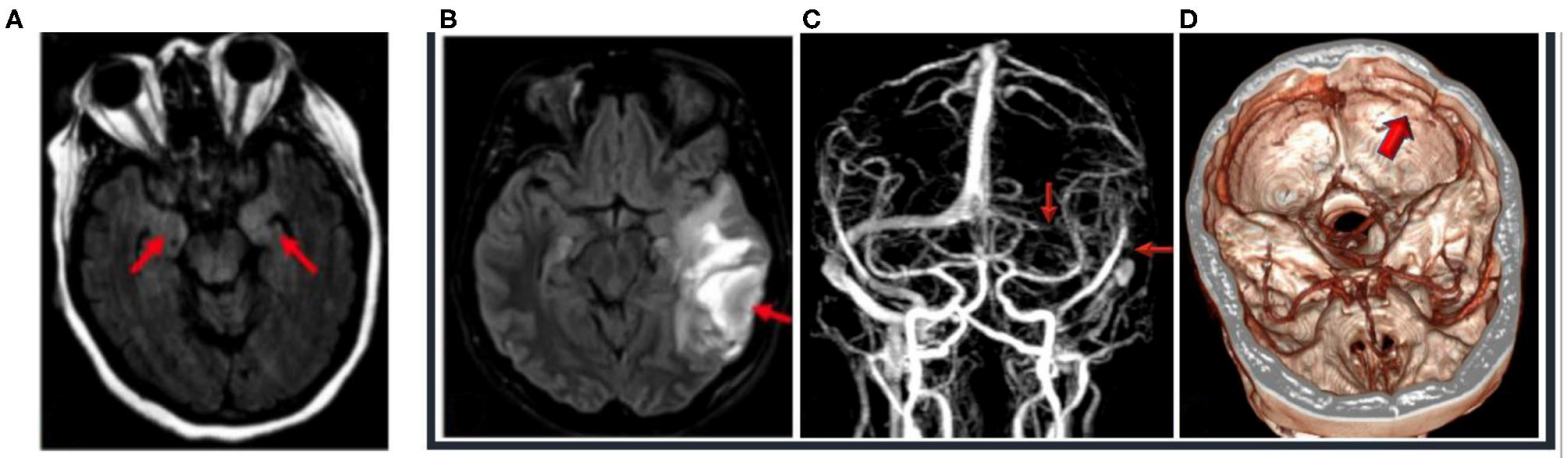
**(A)** Case 1: 48-year-old, COVID-19-positive female with altered mental status; 3-T MRI axial FLAIR image (red arrows) of hyperintense FLAIR in the medial temporal lobe because of altered mental status and seizures, unable to hold still for scan, with resultant motion artifact. **(B–D)** Case 2: 29-year-old, COVID-19-positive female who presented with seizures. **(B)** 1.5-T MRI axial FLAIR (red arrow) of the left temporal lobe with hemorrhagic venous infarction, with the FLAIR hyperintensity consistent with edema and mass effect effacing the left ambient cistern. **(C)** 1.5-T MRI—time-of-flight MRI—with red arrows noting the absent flow void with venous thrombosis of the left transverse and sigmoid sinus and the left internal jugular vein. **(D)** CT venogram with the purple arrow demonstrating absent contrast enhancement and thrombosis of the left transverse and sigmoid sinus.

The patient remained febrile, tachycardic, and tachypneic, requiring intubation for hypoxemic respiratory failure. A 5-day course of high-dose IV methylprednisolone was given for suspected COVID-19-associated central nervous system (CNS) involvement, as well hydroxychloroquine and a single 400-mg dose of tocilizumab. On day 13, the patient was extubated, with significant neurological improvement and resolution of her aphasia. MRI of the brain on day 19 demonstrated T2-weighted (T2) and fluid-attenuated inversion recovery (FLAIR) hyperintensity in the medial temporal lobes bilaterally without restricted diffusion (Figure 1). The patient gradually improved and was discharged on day 25.

### Case 2

A 29-year-old woman with iron deficiency anemia and menorrhagia presented after two GTCS with postictal confusion in the context of preceding fever, cough, and headache for 2 weeks. The initial vital signs were normal, except for tachycardia. While under observation, she suffered another GTCS with right gaze deviation, forced eye opening, tonic limb movements, and loss of awareness. This was aborted with IV lorazepam and levetiracetam. Postictal right-sided paralysis was noted.

The nasopharyngeal COVID-19 PCR test result was positive. Prolactin and D-dimer were notably elevated. Non-contrast CT of the head revealed edema, mass effect, and subcortical hemorrhages in the left temporal and parietal lobes as well as a hyperdense left transverse sigmoid sinus (Figure 1). A CT venogram confirmed left sigmoid and transverse cortical venous thrombosis (CVT), for which IV heparin was commenced. Pulmonary ground-glass opacities were noted on imaging.

EEG demonstrated asynchronous slowing and attenuation over the left hemisphere. CT of the head on day 2 showed evolving left temporal–parietal hemorrhagic infarction with midline shift and partial effacement of the right lateral and third ventricles. The patient developed bilateral abducens palsies and papilledema and was given acetazolamide for increased intracranial pressure. Mentation improved significantly by day 9 as she was able to respond to questions and moved all limbs antigravity, with a mild right facial droop. Hypercoagulable workup revealed anticardiolipin IgM antibodies. The patient was discharged on enoxaparin and levetiracetam.

### Case 3

A 64-year-old woman with a history of hypertension, stage 4 chronic kidney disease, and non-insulin-dependent type 2 diabetes presented after two GTCS, lasting 2 min each, with postictal unresponsiveness. There had been 3 days of preceding headache, vomiting, cough, and malaise. The patient was febrile, tachycardic, and tachypneic, with a blood pressure of 213/100 mmHG. She appeared obtunded with dilated and poorly responsive pupils, right gaze preference, and minimal withdrawal in all limbs to painful stimuli. Under observation, she suffered two additional GTCS and was treated with IV lorazepam, levetiracetam, and fosphenytoin with clinical cessation. Labetalol was given for hypertension.

The laboratory results were significant for thrombocytopenia (77 K/μl), hyponatremia (126 mmol/L), elevated creatinine (2.7 mg/dl), blood urea nitrogen (40 mg/dl), and elevated ferritin. The non-contrast CT of the head and the chest radiograph were unrevealing. The CSF showed four nucleated cells with lymphocytic predominance, while gram stain, culture, and herpes testing were negative. The nasopharyngeal COVID-19 PCR was positive.

**Figure 2:**
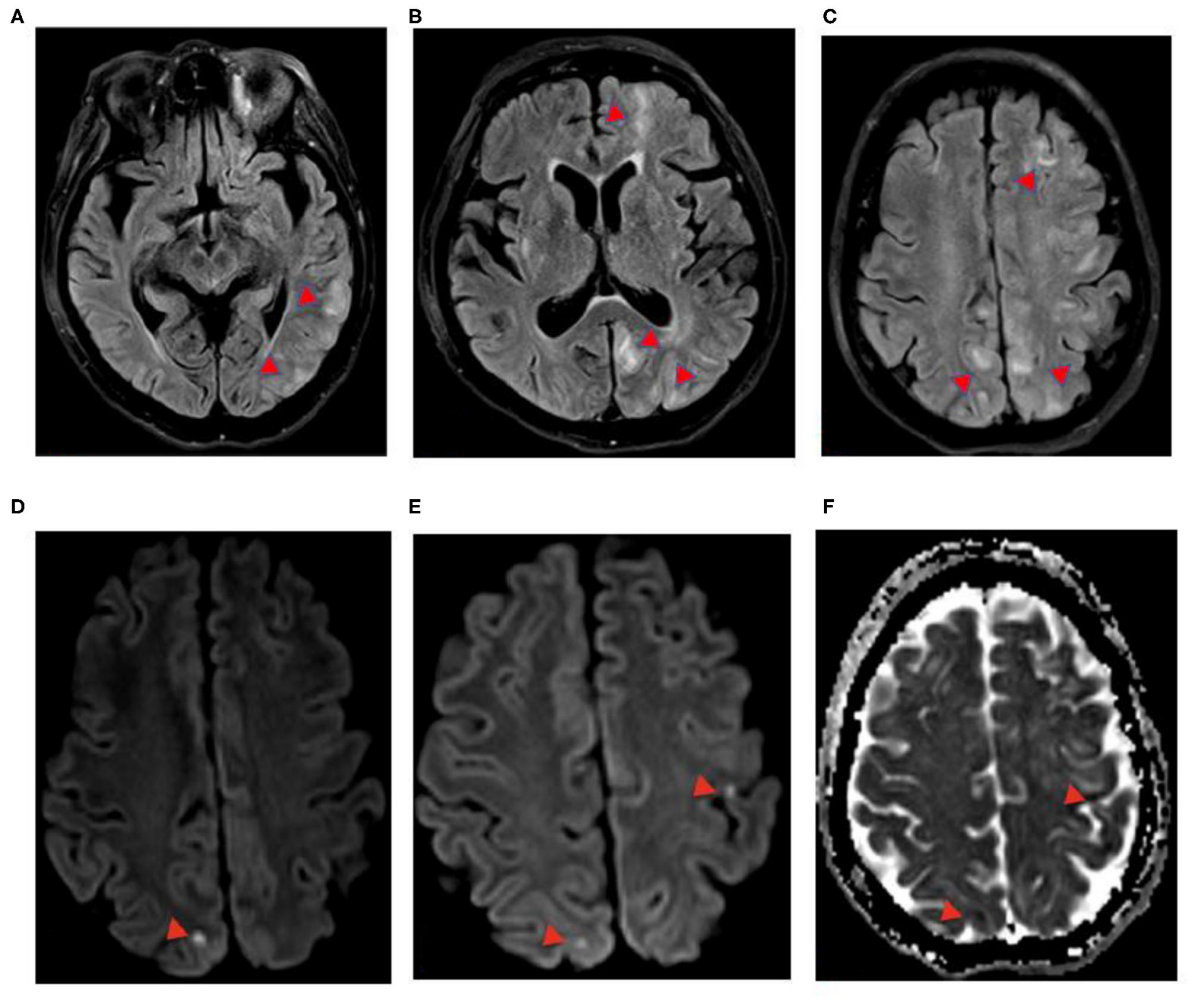
**(A–F)** Case 3: 64-year-old COVID-positive female, axial 3-T MRI. **(A–C)** Axial FLAIR. **(D,E)** Axial DWI. **(F)** Axial apparent diffusion coefficient (ADC). FLAIR hyperintensity in the left was greater than that in the right cerebral hemispheres, with punctate DWI hyperintensity (red arrowheads) with ADC restricted diffusion. **(F)** Left precentral sulcus and right superior medial right parietal lobe, foci of FLAIR, DWI hyperintensity without restricted diffusion, and ADC T2-shine through, concerning posterior reversible encephalopathy in a patient with elevated blood pressure.

EEG was notable for generalized periodic discharges and slowing. Brain MRI without gadolinium, due to kidney dysfunction, showed punctate diffusion positive lesions in the left precentral sulcus and superior right medial parietal subcortical regions as well as patchy T2 prolongation in the left frontal, occipital, and bilateral parietal and cerebellar regions, most consistent with posterior reversible encephalopathy syndrome (PRES) (Figure 2).

The patient remained obtunded without seizures on continuous EEG, with steady improvement of her hyponatremia and blood pressure. Though PRES was of primary concern, a 5-day course of high-dose IV methylprednisolone was given due to consideration of inflammation-mediated seizures associated with COVID-19. The respiratory function was stabilized, and mentation improved by day 10. On day 21, she was discharged home at clinical baseline.

### Case 4

A 68-year-old woman presented for diarrhea, fever, malaise, cough, and shortness of breath and found to be positive for COVID-19 3 days prior. She was febrile, tachycardic, tachypneic, and hypoxemic upon arrival. The chest X-ray showed bilateral lung infiltrates for which she was treated with azithromycin, ceftriaxone, and hydroxychloroquine. The patient decompensated, requiring intubation due to acute respiratory distress syndrome, and was given methylprednisolone and anakinra.

**Figure 3:**
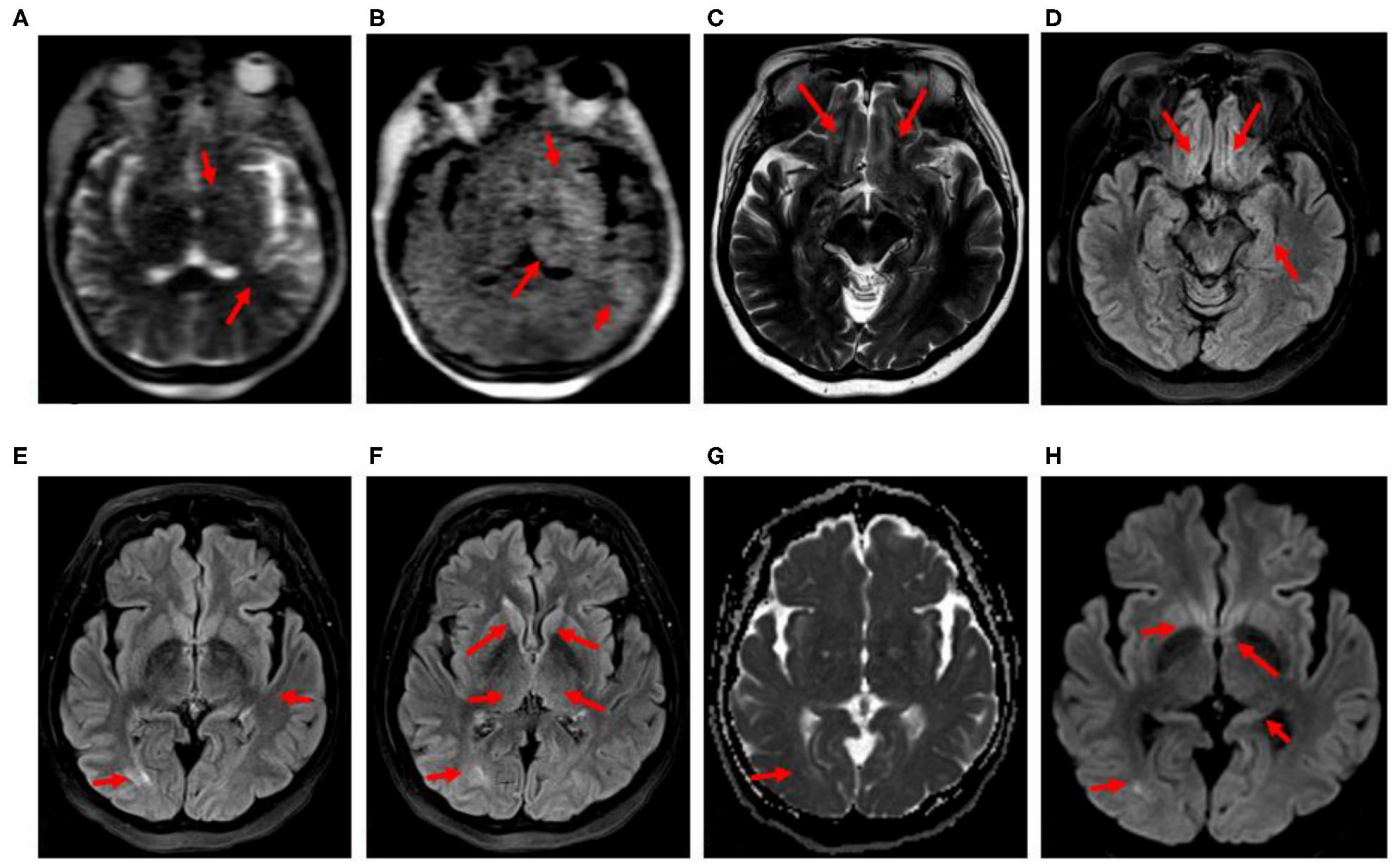
**(A–H)** Case 4: 68-year-old, COVID-positive female. **(A,B)** Hyperfine 0.064-T portable MRI obtained 26 days after admission at the patient's bedside showing focal T2 and FLAIR hyperintensities (red arrows). **(C–H)** 3-T MRI obtained 39 days after admission. **(C)** Axial T2 and **(D)** axial FLAIR hyperintensities (red arrows): left subinsular cortex, lentiform nuclei, thalami, and left temporal lobe. **(E,F)** Axial FLAIR, **(G)** axial ADC, **(H)** axial DWI, T2, FLAIR, and DWI hyperintensities (red arrows): bilateral olfactory tracts, medial temporal lobes including hippocampal tails, caudate heads, fornices and posterior commissural fibers, right posterior lateral temporal lobe without restricted diffusion.

At 2 weeks into her course, the patient remained obtunded off of sedation. The head CT obtained on days 16 and 25 after admission demonstrated decreased attenuation in the left subinsular cortex and the thalamic region. EEG on day 17 showed severe generalized slowing. On day 26, portable 0.06-T MRI (Hyperfine) of the brain without contrast was performed, showing areas of T2 and FLAIR hyperintensity in the left thalamus and lentiform nuclei, though technically limited (Figure 3).

**Figure 4:**
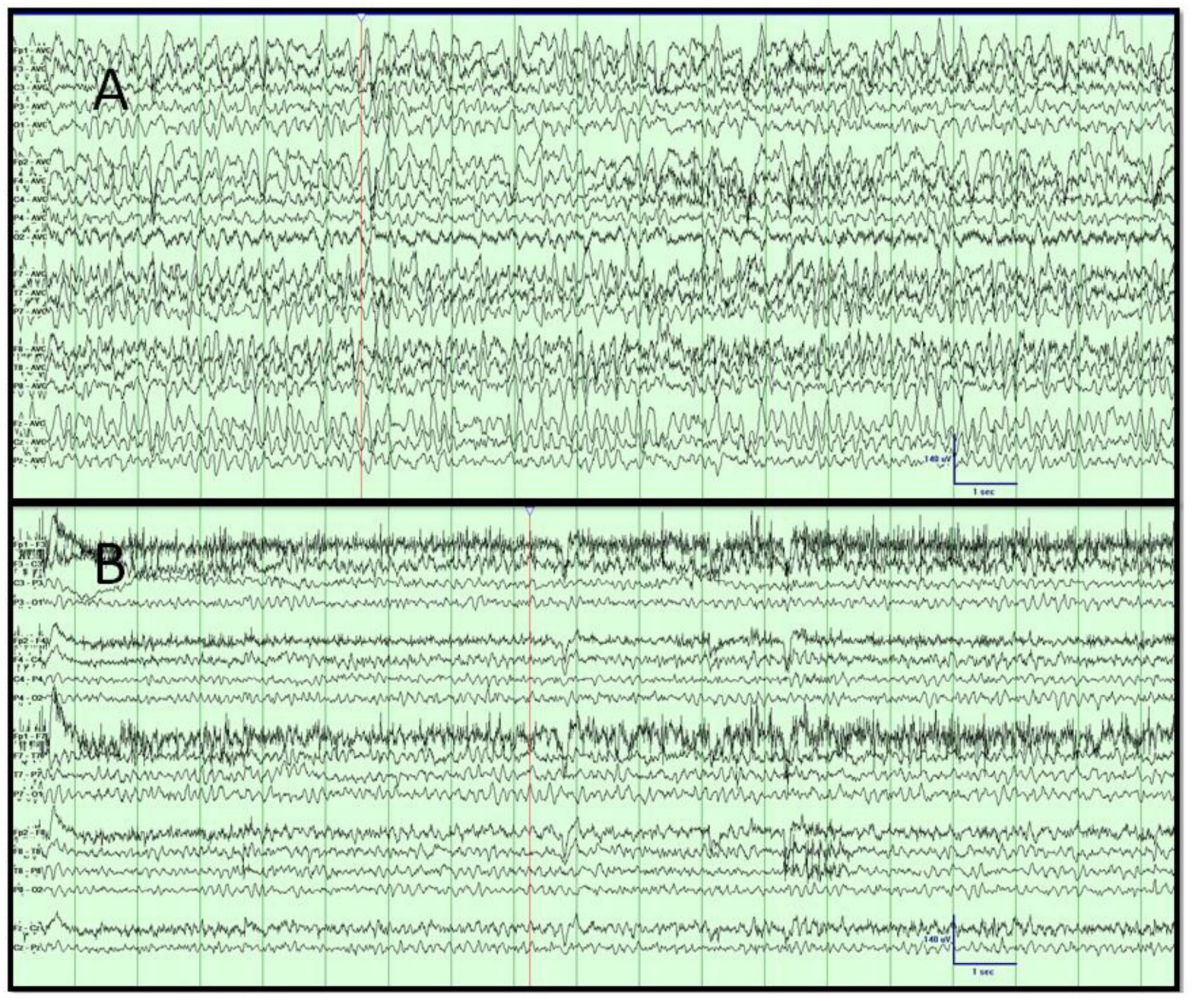
**(A,B)** Case 4. Video EEG results. **(A)** Day 1 showing the high-amplitude rhythmic sharply contoured; intermixed 3- and 6-Hz activity concerning non-convulsive status epilepticus. **(B)** Day 2 with resolution of rhythmic slowing after the administration of lorazepam and levetiracetam, corresponding with improved mentation. Low-frequency filter, 1 Hz; high-frequency filter, 70 Hz; sensitivity, 7 μV/mm; timebase, 30 mm/s.

By day 27, she was extubated but remained confused, with the psychomotor slowed, and unable to follow commands. EEG on day 33 revealed unremitting high-amplitude centrotemporal sharp rhythmic 3–5-Hz activity concerning non-convulsive status epilepticus (NCSE) (Figure 4). Clinical improvement was noted on the following day after the administration of IV lorazepam, levetiracetam, and lacosamide, with EEG transitioning to moderate background, slowing with intermittent left temporal sharp waves. The CSF results on day 36 were normal, including for COVID-19 PCR. A follow-up 3-T contrast-enhanced brain MRI on day 39 showed T2, FLAIR, and diffusion-weighted imaging (DWI) hyperintensity without restricted diffusion along the bilateral olfactory tracts, caudate heads, posterior commissural fibers of the fornices, hippocampal tails, and right temporal lobe (Figure 3). By day 49, the patient was discharged to rehabilitation, alert and capable of conversation, though with moderate persistent cognitive and bilateral proximal weakness.

Summaries of the pertinent clinical details of each case are presented in Table 1.

**Table 1:** Case summaries.

	**Clinical presentation**	**Pertinent neurological findings**	**Seizure semiology**	**Intubated**	**Serum inflammatory markers**	**CSF results**	**EEG findings**	**Radiological findings**	**Possible mechanism of neuronal injury**
CASE # 1	Fever, cough, vomiting, malaise, fever, tachycardia	Global aphasia, bilateral babinski signs	GTCS	Yes	ESR = 9; CRP = 32.6; D-dimer = 279; Fibrinogen = 352; Ferritin= 357.4; Procalcitonin = 1.23	WBCs = 5/uL; Protein = 125 mg/dL; Glucose = 117 mg/dL; Gram Stain, culture, and HSV PCR negative; COVID-19 PCR unavailable	Severe generalized slowing	MRI brain: T2 hyperintensities in the medial temporal lobes	Viral invasion vs. secondary inflammation
CASE # 2	Fever, cough, dyspnea, headache, tachycardia	Encephalopathy, right hemiparesis	FBTCS	No	ESR = 30; CRP = 111.7; D-dimer = 2,876; Fibrinogen = N/A Ferritin= 10.4; Procalcitonin = 0.06	N/A	Asynchronous slowing and left sided attenuation	CT head: edema, mass effect and subcortical hemorrhages in the left temporal and parietal lobes. CTV: left sigmoid and transverse cortical venous thrombosis	CVT
CASE # 3	Headache, vomiting, cough, malaise, hypertension	Encephalopathy	FBTCS	No	ESR = 22; CRP = < 0.1; D-dimer = <150; Fibrinogen = 588; Ferritin= 3,647; Procalcitonin = 0.45	WBCs = 4/uL; Protein = 43 mg/dL; Gram Stain, culture, and HSV PCR negative; COVID-19 PCR unavailable	GPDs	MRI brain: multiple diffusion positive lesions in the left precentral sulcus and right medial parietal regions; patchy T2 prolongation in the left frontal, occipital, and bilateral parietal and cerebellar regions	PRES
CASE # 4	Fever, diarrhea, malaise, cough, dyspnea	Coma, subsequent prolonged encephalopathy	NCSE	Yes	ESR = 77; CRP = 22.8; D-dimer = 1,137; Fibrinogen = 747; Ferritin= 22.8; Procalcitonin = 0.29	WBCs = 1/uL; Protein = 30 mg/dL; Glucose = 73 mg/dL; Gram Stain, culture, and HSV PCR negative; COVID-19 PCR unavailable	High amplitude rhythmic frontotemporal theta, intermixed left temporal sharp waves	MRI Brain = T2, FLAIR and DWI hyperintensity without restricted diffusion along the bilateral olfactory tracts, caudate heads, fornices, hippocampi and right temporal lobe	Secondary inflammation

*Key: GTCS, Generalized tonic-clonic seizures; GPDs, Generalized periodic discharges; FBTCS, Focal to bilateral tonic-clonic seizures; NCSE, Non-convulsive status epilepticus; PRES, Posterior reversible encephalopathy syndrome; MRI, Magnetic resonance imaging; ESR, Erythrocyte sedimentation rate; CRP, C-Reactive protein; WBCs, White blood cells; HSV, Herpes simplex virus; PCR, Polymerase chain reaction; COVID-19, SARS-CoV-2; CVT, Cortical venous thrombosis; CT, Computed tomography; N/A, Not available. Normal laboratory values: ESR = 4–25 mm/h; CRP <5 mg/L; D-dimer <230 ng/mL; Fibrinogen = 300–500 mg/dL; Ferritin = 15–150 ng/mL; Procalcitonin = 0.02–0.1 ng/mL; CSF Protein = 15–40 mg/dL; CSF Glucose = 40–70 mg/dL; CSF WBCs = 0–5 cell/uL*.

## Discussion

The cases above describe four scenarios of seizures occurring in association with COVID-19, illustrating the remarkable variety of neurological complications accompanying this disease. Seizures in this report may occur by differing mechanisms, including in the context of inflammatory changes in the brain as demonstrated by neuroimaging or vascular abnormalities related to the effects of SARS-CoV-2 on hypercoagulability and endothelial cell dysfunction.

In summary, the first case presented with seizures during the early acute phase of infection, with elevated inflammatory markers, and FLAIR changes in the temporal region on MRI indicative of focal inflammation (Figure 1A). Lumbar puncture did not reveal an evidence for encephalitis. In addition to antiseizure medications, the patient responded well to immunotherapy, with good neurological outcome, supporting a hypothesis of excessive innate immune activation. In case 2, the seizures resulted from acute CVT in the left transverse and sigmoid sinus, resulting in a left temporal lobe venous infarct (Figures 1B,D), highlighting a coagulopathic state associated with COVID-19. The seizures observed in case 3 manifested in the setting of a patient with elevated blood pressure and end-stage kidney disease with PRES, raising concern for COVID-19 effects on endothelial cell function and vascular permeability. This patient demonstrated classic findings of increased T2, DWI, and FLAIR hyperintensity without restricted diffusion in the setting of elevated blood pressure (Figures 2A–F) and with a few punctate foci of restricted diffusion and ischemic change. The association of PRES as a neurologic complication of COVID-19 has been recently documented in the literature (15–18). The final patient was determined to have seizures later in her course, along with persistent altered mentation and radiographic findings suggestive of inflammatory changes and concern for immune-mediated necrotizing encephalopathy. Early portable magnetic resonance imaging using a Hyperfine MRI at the bedside, as the patient was too unstable to come down to the scanner (Figures 3A,B), demonstrated thalamic T2 and FLAIR hyperintensity, with follow-up conventional magnetic resonance imaging 2 weeks later showing bilateral hippocampal, forniceal, sub-insular, and orbitofrontal T2 and FLAIR abnormalities. Initiating antiseizure medications led to significant electrographic improvements and eventual delayed clinical improvement.

A few recent case reports are published in the literature describing acute symptomatic repetitive seizures in the setting of COVID-19. In an early report, the patient had positive CSF COVID-19 PCR and mesial temporal and lateral ventricular signal hyperintensities on MRI indicative of encephalitis (3). Additional rare cases may present with lymphocytic pleocytosis (19).

*De novo* focal seizures including status epilepticus in the absence of other medical comorbidities, neuroimaging, or lumbar puncture abnormalities are also described (7, 9–12, 20). New-onset seizures are reported in medically complex elderly patients with COVID-19; however, these were in the setting of multiorgan failure, metabolic derangements, and, in certain patients, superimposed sepsis or hypotension as complicating factors, and unfortunately, MRI or lumbar puncture results were often unavailable (4, 7, 8). Seizure exacerbation and status epilepticus may also be exacerbated in patients with preexisting epilepsy and focal lesions, where COVID-19 infection may have lowered the seizure threshold (5, 21).

In a series of 214 consecutive patients with COVID-19 from China, only one individual experienced a convulsion, although 16 additional patients were reported with unspecified impaired consciousness (1). An additional retrospective multicenter study of 304 consecutive patients with COVID-19 reported only two cases of seizure-like events (2). One patient presented with facial deviation and acute anxiety, while the other experienced myoclonus with electrolyte imbalance. Eight additional patients were described as encephalopathic or comatose. Notably, in both publications, EEG was not performed due to concerns regarding infection control.

Although clinical seizures are infrequently encountered with COVID-19, NCSE may still need to be excluded by EEG in patients with unexplained altered mentation. Investigators recognize encephalopathy as a common occurrence among severely ill patients with COVID-19; however, concurrent EEG data are often unavailable, and further research is required to understand the true incidence of electrographic seizures in patients with SARS-CoV-2 infection. Relatively small cohorts of patients with COVID-19 and EEG are published to date, with the patients in these studies only undergoing routine duration or reduced montage EEG recording, for the reasons discussed above, and less frequently continuous EEG monitoring. Several authors observed sporadic epileptiform abnormalities and periodic patterns of concern, including generalized periodic discharges, along with more expected patterns of generalized slowing (22–26). Due to centers taking precautions to avoid transmission risk by reducing staffing and utilization of EEG, as well as obtaining imaging, cases of electrographic seizures and NCSE may be less likely to be diagnosed and adequately imaged. While the safety and risks of infection must be weighed carefully, patients may potentially benefit from the use of EEG and neuroimaging to recognize potentially treatable seizures.

The speculative mechanisms responsible for the neurological manifestations of SARS-CoV-2 are grouped broadly into direct viral invasion and indirect effects *via* systemic inflammation, coagulopathic states, endotheliopathy, and homeostatic disruptions, including metabolic derangements due to hypoxia and organ failure.

Given the genetic semblance between SARS-CoV and SARS-CoV-2 and the shared manner of cellular entry *via* receptors for angiotensin-converting enzyme II (ACE2), neurotropism may be similar (27). Previous studies have demonstrated SARS-CoV antigens and RNA within human neurons, supporting a hypothesis of neuroinvasion in SARS-CoV-2 (28). One proposed route of direct cell invasion involves receptors for ACE2, found on the surface of glia and endothelial cells, allowing for distinct ports of CNS entry (29, 30). CNS penetration may involve trans-axonal transport along adjacent glia or hematogenous spread. Invasion *via* cells expressing ACE2 in the olfactory tract may lead to encephalitis associated with seizures and MRI signal changes indicative of edema and inflammation (3).

The upregulation of proinflammatory cytokines may also play a role in para-infectious immune-mediated neurological complications, such as demyelinating disease, acute necrotizing encephalopathy, or Guillain–Barre syndrome in COVID-19 (31–36). Endothelial dysfunction and disruption of the integrity of the blood–brain barrier may result in cerebral antigen exposure and increased susceptibility to abnormal adaptive immunity. Similar neuroimmunological manifestations are reported with other coronaviruses, and seizures have been reported in this context (HCoV-OC43, SARS CoV, and MERS) (14, 30). These neurological presentations highlight the need for brain imaging in encephalopathic patients with COVID-19, particularly those with focal neurological deficits or seizures, which may reflect aberrant and excessive autoimmune phenomena. The neuroimaging findings in COVID-19 are extremely heterogenous and may vary based on the severity of infection (37). Immunotherapy, such as corticosteroids, targeted molecular therapies such as monoclonal antibodies, IV immunoglobulins, or convalescent plasma for patients with these manifestations may be potentially beneficial, though more systematic research is required (36, 38–40). During their course of treatment early on in the New York area pandemic, three patients in our series received methylprednisolone, two received hydroxychloroquine, one received tocilizumab, and one was administered anakinra. The treatments for COVID-19 remain highly variable at this time, and therapies specifically for neurological complications lack consensus. While potentially helpful for cytokine storming, data have subsequently called into question the early recommendations to utilize hydroxychloroquine in hospitalized COVID-19 patients due to safety concerns (36). While corticosteroids have significant anti-inflammatory effects, caution is likewise advised to avoid the potential delay of viral clearance or other systemic complications, and timing and dosing of steroids may need to be considered carefully on a case-by-case basis for more severe cases of neuroinflammation (41). Research continues in support of drugs targeting cytokines *via* the IL-6 receptor, such as tocilizumab, and IL-1 receptor, such as anakinra (36).

Endothelial dysfunction may contribute to vascular events such as stroke (35). Viral cellular invasion *via* ACE2 expressed on the surface of glia, endothelial cells, myocardium, and arterial smooth muscle may predispose patients to thromboinflammation (42). Increases in systemic proinflammatory cytokines observed in COVID-19 such as IL-6, IL-10, and TNF-α are associated with hypercoagulability and microthrombi (43, 44). Elevated D-dimer levels are associated with thrombus formation and more severe infection and mortality (42). In one study, 6% of patients with COVID-19 were found to have acute strokes, theoretically predisposing them to a higher incidence of seizures (1). Nearly 3% of non-COVID-19 adults with ischemic stroke suffer early seizures, while the rates of seizures in CVT are as high as 34% (45, 46). CVT, as described in case 2, in COVID-19 has been reported, and given seizures as a common presenting feature, one should remain vigilant for this entity in the appropriate clinical context (47).

PRES in the setting of COVID-19 may similarly involve elevated cytokines due to systemic hyperinflammation, leading to endothelial activation and increased cerebrovascular permeability and edema. In the patient presented, underlying hypertension and renal disease may have been predisposing factors exacerbated by COVID-19, with additional cases of PRES in COVID-19 also reported (15, 16, 18, 48).

Acute seizures may also occur as a result of other systemic complications of COVID-19 resulting from hypoxia and inflammation, including multiorgan failure and associated metabolic disorders such as uremia, electrolyte abnormalities, and potential drug toxicity (14).

Seizures are a serious neurological manifestation of COVID-19. Their occurrence may be a concerning indicator of direct viral CNS involvement, autoimmune-mediated inflammation of the brain, neurovascular compromise, or cerebral autoregulatory abnormalities for which clinicians may need greater awareness of as potentially grave complications of SARS-CoV-2. Appropriate early diagnosis and treatment, with interventions such as antiseizure medications, immunotherapy, or modulation of blood pressure, may improve the outcome in these cases. Continued research is required to understand the true prevalence of electrographic seizures in a COVID-19-related neurological illness in a larger case series and to correlate those findings with radiographic abnormalities and effects on clinical outcome.

## Data Availability Statement

The original contributions presented in the study are included in the article/supplementary material, further inquiries can be directed to the corresponding author.

## Ethics Statement

Written informed consent was obtained from the individual(s) for the publication of any potentially identifiable images or data included in this article.

## Author Contributions

SH, AB, UM, AS, and AH were responsible for the initial conception and draft of this manuscript. CK was responsible for neuroimaging descriptions and image preparation. CK, RK, and SN were responsible for guidance and editing of the manuscript. All authors contributed to the article and approved the submitted version.

## Conflict of Interest

The authors declare that the research was conducted in the absence of any commercial or financial relationships that could be construed as a potential conflict of interest.
